# Injectable Scaffolds for Adipose Tissue Reconstruction

**DOI:** 10.3390/gels12010081

**Published:** 2026-01-17

**Authors:** Valeria Pruzzo, Francesca Bonomi, Ettore Limido, Andrea Weinzierl, Yves Harder, Matthias W. Laschke

**Affiliations:** 1 Institute for Clinical and Experimental Surgery, Saarland University, PharmaScienceHub (PSH), 66421 Homburg, Germany; pruzzovaleria@gmail.com (V.P.); francescabonomi.bonnie@gmail.com (F.B.); limidoettore@gmail.com (E.L.);; 2 Department of Surgery, Ospedale Beata Vergine Mendrisio, Ente Ospedaliero Cantonale (EOC), 6850 Mendrisio, Switzerland; 3 Department of Plastic Surgery and Hand Surgery, University Hospital Zurich, 8091 Zurich, Switzerland; 4 Department of Plastic, Reconstructive and Aesthetic Surgery and Hand Surgery, Centre Hospitalier Universitaire Vaudois (CHUV), 1011 Lausanne, Switzerland; yves.harder@chuv.ch; 5 Faculty of Biology and Medicine, University of Lausanne (UNIL), 1011 Lausanne, Switzerland

**Keywords:** injectable scaffold, hydrogel, adipose tissue engineering, soft tissue reconstruction, fat graft retention, extracellular matrix, decellularized adipose tissue

## Abstract

Autologous fat grafting is the main surgical technique for soft tissue reconstruction. However, its clinical use with more extended volumes is limited by repeated procedures due to the little possibility of banking tissue, donor-site morbidity and unpredictable graft resorption rates. To overcome these problems, adipose tissue engineering has focused on developing injectable scaffolds. Most of them are hydrogels that closely mimic the biological, structural and mechanical characteristics of native adipose tissue. This review provides an overview of current injectable scaffolds designed to restore soft tissue volume defects, emphasizing their translational potential and future directions. Natural injectable scaffolds exhibit excellent biocompatibility but degrade rapidly and lack mechanical strength. Synthetic injectable scaffolds provide tunable elasticity and degradation rates but require biofunctionalization to support cell adhesion and tissue integration. Adipose extracellular matrix-derived injectable scaffolds are fabricated by decellularization of adipose tissue. Accordingly, they combine bio-mimetic structure with intrinsic biological cues that stimulate host-driven adipogenesis and angiogenesis, thus representing a translatable “off-the-shelf” alternative to autologous fat grafting. However, despite this broad spectrum of available injectable scaffolds, the establishment of clinically reliable soft tissue substitutes capable of supporting large-volume and long-lasting soft tissue reconstruction still remains an open challenge.

## 1. Introduction

Soft tissue reconstruction—be it extended surface defects or organ defects such as in the breast—represents a major challenge for plastic and reconstructive surgeons. Autologous fat grafting is currently one of the options for volumetric restoration. Clinically, this approach is applied to correct deformities after oncologic resections, traumatic injuries and congenital anomalies [1]. Its autologous and minimally invasive nature reduces the risk of immunogenic reactions and foreign body responses, while resulting in esthetically natural outcomes. Despite these benefits, the technique is limited by the need of repeated surgeries to harvest and recreate volume, the regulatory issues regarding banking autologous fat, donor-site morbidity and eventually unpredictable fat graft resorption [2,3,4].

Once transplanted, adipocytes in the grafted fat are solely nourished by passive diffusion of oxygen from the surrounding host tissue until neovascularization of the transplanted tissue occurs. This diffusion distance is limited to approximately 300 µm from the supplying blood vessels [5,6]. Accordingly, the central region of the graft is particularly susceptible to hypoxia and undergoes necrotic cell death [6]. Consequently, graft retention remains inconsistent with reported resorption rates of 18–80% [7]. Further, long-term volume retention depends on many patient factors, including significant weight changes [8,9]. This has motivated the development of novel approaches that promote early graft vascularization and survival. Although enrichment of fat grafts with adipose-derived stem cells (ASCs), platelet-rich plasma or angiogenic factors has shown potential, no standardized protocol has yet successfully translated into clinical practice [10,11]. This is also because many patients that may benefit from these procedures are oncological patients, for example, breast cancer patients.

Over the past decades, adipose tissue engineering has emerged as a promising strategy to overcome the above-mentioned limitations. By combining cells, scaffolds and bioactive elements, it aims to mimic the native microenvironment of adipose tissue and to guide tissue regeneration. In this context, injectable scaffolds have gained significant attention due to their ability to homogenously fill up irregular defects and to support adipogenesis and neovascularization within a three-dimensional framework [12,13]. Moreover, their ease of handling and their minimally invasive application, even outside the operating room, make them a valid option for clinical translation [14].

Injectable scaffolds can either serve as stand-alone matrices for host tissue ingrowth or as carriers for ASCs and growth factors. Several types of injectable scaffolds, including natural, synthetic and adipose extracellular matrix (ECM)-derived biomaterials, have been investigated so far. Each of them offers unique properties in terms of biocompatibility, mechanical performance and bioactivity. This review provides a comprehensive overview of these injectable scaffolds for soft tissue reconstruction, highlighting translational opportunities and future directions.

## 2. Adipose Tissue Engineering

A major goal of adipose tissue engineering is to develop tissue substitutes that closely mimic not only the biological but also the structural and mechanical characteristics of native adipose tissue by means of scaffolds, thereby providing a suitable microenvironment for soft tissue reconstruction and regeneration [15,16]. Native adipose tissue consists of mature adipocytes and the stromal vascular fraction (SVF), which is embedded in an ECM that is rich in collagen, laminin, fibronectin, elastin, glycosaminoglycans and growth factors. The dynamic crosstalk between cells and the ECM, mediated by integrin-dependent mechano-transduction, is a key aspect of adipose tissue engineering. Indeed, this interaction regulates the differentiation and proliferation of ASCs and preadipocytes, supporting adipogenesis for up to ~3 months post-grafting [17].

The ideal injectable scaffold should be self-gelling, biocompatible, capable of incorporating cells and bioactive molecules, and able to promote adipogenesis without requiring extensive manipulation [12]. A scaffold viscoelasticity in the range of 2–10 kPa has been shown to mimic the mechanical properties of native adipose tissue and, thus, to favor adipogenic differentiation [18]. Moreover, scaffold degradation should occur in parallel with new tissue formation and remodeling, providing structural support during the initial 3-month phase of active adipogenesis and the subsequent 12-month period of adipose tissue maturation after grafting [12,19]. Thus, precise tuning of the stiffness and degradation kinetics of scaffolds is crucial to create an ideal microenvironment for cell survival and vascular ingrowth.

Various injectable biomaterials have been investigated as scaffolds in the context of adipose tissue engineering. These include natural, synthetic and adipose ECM-derived biomaterials. Most of them are hydrogels, which provide a three-dimensional, cross-linked hydrophilic network capable of retaining ECM components and ASCs [13]. Natural hydrogels are composed of single components of animal or plant ECM. They support cell adhesion, differentiation and proliferation, while providing excellent biocompatibility. However, they degrade rapidly and lack mechanical strength, often requiring the addition of other natural or synthetic biopolymers. By contrast, synthetic hydrogels provide tunable degradation rates but do not naturally support cell adhesion. As a consequence, material modifications are often necessary to enhance their bioactivity [20]. Adipose ECM-derived hydrogels are produced by decellularizing adipose tissue. This allows the preservation of biochemical properties and ultrastructural features of the native ECM without the need for additional manipulation [21]. For instance, Young et al. [22] reported that decellularized human lipoaspirates retain a complex, adipose tissue-specific composition of collagen isoforms, laminin, fibronectin and sulfated glycosaminoglycans. Although decellularization reduced the concentration of these components compared to native tissue, this assortment of biochemical cues still mimicked the microenvironment of adipose tissue. Uriel et al. [23] generated hydrogels from decellularized subcutaneous fat of donor rats, which polymerized into fibrous networks with a scale and architecture similar to native tissue. Taken together, each type of injectable scaffold offers a distinct balance between biological activity and mechanical properties (Table 1). Accordingly, specific clinical approaches for soft tissue reconstruction may require different scaffold types as outlined in the following sections.

### 2.1. Natural Injectable Scaffolds

Natural injectable scaffolds primarily consist of ECM-derived biopolymers, including collagen, chitosan, elastin, fibrin and hyaluronic acid (HA). In 1981, the first natural polymer to receive approval from the U.S. Food and Drug Administration (FDA) as a dermal filler was type I collagen (Zyderm^®^, Inamed Corporation, Santa Barbara, CA, USA). In 2003, the advent of Restylane™ (Galderma, Zug, Switzerland), a cross-linked HA-based filler, heralded a new era for soft tissue augmentation. This landmark was subsequently followed by the introduction of several HA-based injectable fillers (Table 2), clinically implemented for soft tissue contouring and volumization of breast and face [24]. Despite their clinical success, these products are often expensive and exhibit limited longevity, making them unsuitable for large-volume and durable soft tissue reconstructions. In fact, most commercially available fillers undergo resorption within a period ranging from several weeks to one year, depending on their formulation and the implantation site [25]. Consequently, contemporary adipose tissue engineering research increasingly focuses on the design of natural injectable scaffolds capable of maintaining long-term stability and integration within the host tissue.

To achieve sustained functionality and volume retention of natural hydrogels, a range of strategies have been explored. One widely adopted approach is the incorporation of stem cells or preadipocytes into the hydrogels. For instance, Torio–Padron et al. [26] demonstrated that a fibrin hydrogel combined with 1 × 10^6^ preadipocytes achieved approximately 50% retention of the grafted volume (1 mL) 4 weeks following subcutaneous injection in nude mice. By contrast, the fibrin hydrogel alone underwent complete resorption within 3 weeks. In line with this finding, Huang et al. [27] reported that a HA hydrogel combined with ASCs maintained a significantly higher graft volume 8 weeks post-implantation in nude mice when compared to ASCs-only or scaffold-only controls. In another study, mesenchymal stem cell (MSC)-loaded chitosan microspheres enhanced adipogenic differentiation and resulted in superior volume retention relative to either scaffold-alone or MSCs-alone treatments following injection into malar defects in rabbits [28]. This phenomenon is commonly attributed to a dual action of natural hydrogels combined with such adipose cellular components. On the one hand, the bioactivity of the natural injectable scaffold actively supports cell differentiation and proliferation. On the other hand, the degradation of the scaffold over time allows progressive replacement by newly formed adipose tissue [27]. Consequently, the degradation kinetics of injectable scaffolds is of critical importance. Excessively rapid degradation compromises the scaffold’s ability to provide sufficient mechanical support and biochemical cues, whereas too slow degradation may hinder tissue ingrowth [29].

The need to synchronize scaffold degradation with new tissue formation has driven growing interest in crosslinking strategies for natural injectable scaffolds [30]. Crosslinking enables precise tuning of both scaffold degradation rate and mechanical stiffness, tailoring the scaffold to the specific demands of the host environment. Over the years, a broad range of crosslinked natural injectable hydrogels has been developed [31]. For example, Louis et al. [32] engineered a fibrin–collagen crosslinked hydrogel preconditioned in vitro with human ASCs, adipocytes and endothelial cells. Upon subcutaneous implantation in mice, the scaffold showed significantly higher cell survival rate (~84%) and better volume retention at 3 months when compared to conventional fat grafts [32]. More recently, Challapalli et al. [33] incorporated ASCs into HA hydrogel crosslinked in situ using horseradish peroxidase (HRP) and hydrogen peroxide (H_2_O_2_). This enzymatic crosslinking strategy enhanced the mechanical stability, slowed the scaffold degradation rate and maintained a scaffold elasticity of 6–8 kPa. Moreover, it enabled controlled polymerization allowing the hydrogel to conform precisely to the shape of the surgical defect. Accordingly, in a murine mastectomy model this HA hydrogel supported high cell viability, enhanced adipogenesis and sustained graft volume retention over 4 weeks post-implantation, outperforming both scaffold-only and ASCs-only controls [33].

The long-term success of fat grafts seems also to rely on scaffold architecture. Yao et al. [34] developed injectable hydrogel microspheres by emulsifying a collagen I/alginate mixture via a high-voltage microfabrication device. The resulting microspheres (diameter: ~250–400 μm) were then cultured with human ASCs over 4 weeks, showing great shape-retention and a 39% increase in size after 90 days in vitro. Thereafter, the “fat lobule-like” microspheres were injected subcutaneously into nude mice. Four weeks post-injection, vascularized adipose tissue had formed with significantly more host blood vessels anastomosing with the graft and a higher volume retention rate when compared to a non-microsphere injectable scaffold. This suggests that mimicking the lobular structure of native adipose tissue enhances integration and functionalization of the injected hydrogel [34]. The microsphere approach has also been used for chitosan [28] and chitosan–alginate [35] scaffolds with promising results in terms of graft volume retention.

**Figure 1:**
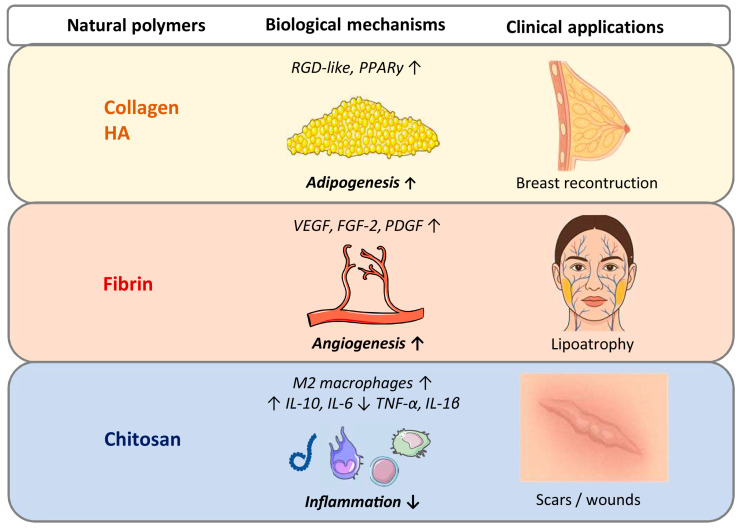
Potential clinical applications of injectable scaffolds based on natural polymers, i.e., collagen, hyaluronic acid, fibrin and chitosan, with different biological activities. FGF = fibroblast growth factor; HA = hyaluronic acid; IL = interleukin; PDGF = platelet-derived growth factor; PPARγ = peroxisome proliferator-activated receptor gamma; TNF = tumor necrosis factor; VEGF = vascular endothelial growth factor; ↑ = increased; ↓ = reduced.

From a clinical perspective, the intrinsic biological properties of each natural polymer distinctly influence cellular behavior and tissue remodeling, thereby suggesting specific application potentials (Figure 1). Both collagen- and HA-based injectable scaffolds seem to favor adipogenesis over angiogenesis. Indeed, by exposing RGD-like binding sites capable of engaging integrins and activating adipogenic signaling pathways, such as the peroxisome proliferator-activated receptor gamma (PPARγ), they promote adipogenesis [33,36]. This is particularly relevant for large defects, such as breast reconstruction. Accordingly, Puls et al. [37] demonstrated that an in situ-forming oligomeric collagen I hydrogel injected into a mastectomy cavity in pigs can support adipogenesis and mammary duct regeneration over 16 weeks. The scaffold maintained its volume and elicited no inflammatory response, even after radiation therapy, making it highly suitable for oncologic reconstructions [37]. Similarly, HA–ASCs hydrogels promoted adipogenesis and volume retention in a murine mastectomy model [33]. However, no radiation therapy was performed in this model. Fibrin-based injectable scaffolds, in turn, exhibit a strong intrinsic angiogenic activity by supporting endothelial cell migration and stimulating the release of endogenous growth factors, such as vascular endothelial growth factor (VEGF), fibroblast growth factor (FGF)-2 and platelet-derived growth factor (PDGF) [38]. However, they exhibit a relatively low volume retention, making them particularly unsuitable for large-volume reconstructions. On the other hand, they might be suitable for smaller and highly vascularized soft tissue defects, such as those in the facial region. Moreover, when combined with SVF or ASCs, these scaffolds further enhance early capillary network formation [39]. Chitosan-based injectable scaffolds, though less extensively investigated, offer promising features for minimally invasive applications. Their thermosensitive formulations enable in situ gelation without the need for chemical crosslinkers [40,41]. Furthermore, their intrinsic surface chemistry promotes the polarization of macrophages toward the regenerative M2 phenotype and regulates cytokine release (i.e., increasing interleukin (IL)-6 and IL-10 expression and decreasing IL-1β and tumor necrosis factor (TNF)-α expression), controlling inflammation [42,43]. Owing to these immunomodulatory properties, chitosan-based systems may be particularly advantageous for the treatment of soft tissue contour restoration particularly in inflamed or fibrotic environments, such as atrophic scars, wounds or traumatic defects.

Taken together, natural injectable scaffolds offer the intrinsic advantage of biocompatibility and bioactivity, while their degradation kinetics and tissue integration outcomes remain unpredictable. When appropriately engineered, these materials offer a minimally invasive, biologically favorable platform for soft tissue regeneration. However, further investigations are needed to assess long-term volume stability, host immune response and scaffold remodeling dynamics. Of note, the current absence of direct comparative clinical studies across all different natural injectable scaffold types limits the ability to establish clear guidelines for material selection in specific surgical contexts. Addressing these gaps would be essential to broadly implement natural injectable scaffolds into clinical practice.

### 2.2. Synthetic Injectable Scaffolds

**Figure 2:**
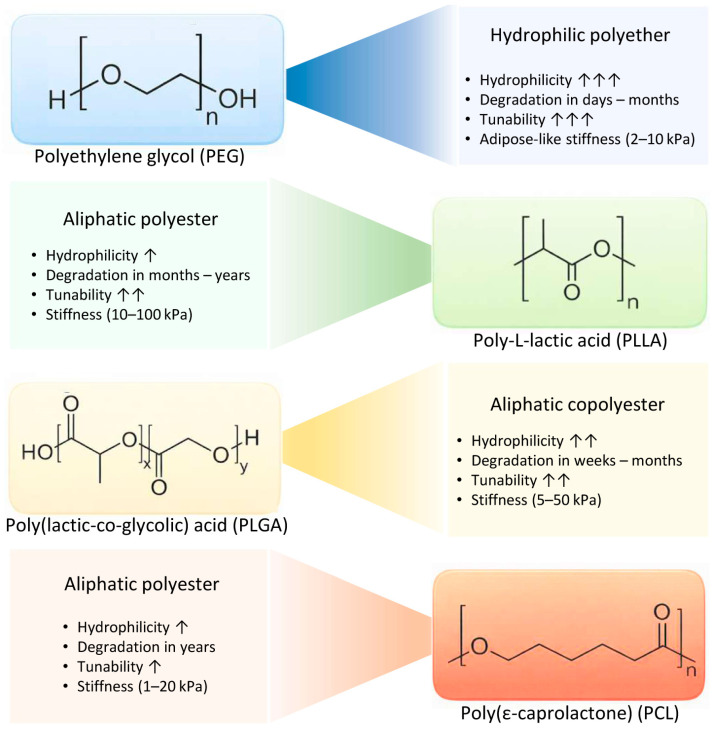
Synthetic injectable scaffolds: classes and properties. ↑ = low; ↑↑ = moderate; ↑↑↑ = high.

Synthetic injectable scaffolds commonly consist of polyethylene glycol (PEG), poly–L–lactic acid (PLLA), poly(lactic-co-glycolic) acid (PLGA) and poly(ε-caprolactone) (PCL) (Figure 2). The main advantage of these materials is their tunable stiffness and degradation rates. One of the main drawbacks is the lack of intrinsic bioactivity. Modifications are therefore necessary to enable cell adhesion and infiltration [44]. For example, PEG–peptide hydrogels can be biofunctionalized via conjugation with laminin-derived adhesion peptide (YIGSR) before seeding them with preadipocytes. The result is an injectable scaffold closer to the mechanical properties and bioactivity of the ECM, allowing cell ingrowth [45]. Moreover, biodegradable polyester microspheres (PLGA, PLLA and PCL) have been used as injectable cues that can mimic the adipose tissue architecture. Spheres with a size of ~40 µm showed the best support of cell attachment without being phagocytosed by macrophages and provided inter-sphere spaces for adipose tissue ingrowth [46]. PLGA degrades over weeks to months, depending on the additives modifying its behavior (i.e., incorporating basic magnesium hydroxide can buffer acidic degradation products and reduce inflammation). PLLA and PCL microspheres function similarly, but degrade more slowly over years, providing longer scaffold presence [46,47]. Thermo-responsive copolymers of PCL with PEG gel rapidly at 37 °C, enabling minimally invasive delivery and in situ scaffold formation via physical micelle aggregation [48].

Short-term adverse effects following the subcutaneous injection of synthetic fillers in humans include localized erythema, ecchymosis or edema, while long-term complications include fibrosis and the formation of subcutaneous nodules [46,49]. To address these issues, researchers have developed hybrid (synthetic and natural) scaffolds to provide mechanical support, biocompatibility and regenerative signals for stable volume restoration. For example, Lee et al. [50] developed a double network PEG–collagen hydrogel that exhibits both suitable mechanical strength and cell adhesiveness and, thus, enhances adipogenesis in vitro. Likewise, Henn et al. [51] showed that a PEG–HA hydrogel improves the vascularization of loaded fractionated adipose tissue when compared to PEG alone, with a stronger infiltration of progenitor cells and regenerative macrophages over 21 days in rats. Furthermore, ECM–PEG composite hydrogels promoted human ASC viability and adipogenic differentiation in vitro when compared to PEG alone, providing a niche for stem cells when implanted into mice [52,53]. In these hydrogels, a concentration of 1% adipose-derived ECM resulted in the highest level of adipogenic differentiation relative to 0.01% and 0.1%, emphasizing the significant contribution of ECM components to the biological performance of this synthetic scaffold [52]. Similarly, another study demonstrated that PLGA–acellular adipose matrix (AAM) microspheres have a greater ability to retain graft volume and improve vascularization when compared to conventional fat grafts after implantation into mice [54]. These examples indicate that the use of such hybrid scaffolds aims to mimic the bioactivity of natural ECM while maintaining the mechanical stability and longevity typical of synthetic polymers.

Overall, synthetic and hybrid scaffolds are proven to stimulate adipose regeneration and new tissue formation both in vitro and in vivo. Clinically, PLLA fillers (e.g., Sculptra^®^, Galderma, Zug, Switzerland) and PCL microsphere fillers (Ellansé^®^, Sinclair Pharma, London, UK) are FDA-approved cosmetic fillers that remain stable for up to ~2 years [55]. Both fillers act by stimulating collagen neogenesis, making them particularly suitable for facial volume restoration, where collagen is resorbed over time. A next-generation filler from China (CureWhite^®^, IMEIK Technology, Beijing, China) combines PLLA–PEG copolymer microspheres within a crosslinked HA gel, resulting in significant improvements in face contouring after 6 and 12 months and offering a good biocompatibility [56,57]. However, despite significant advances in polymer chemistry and biomaterial engineering, no synthetic injectable scaffold has yet been successfully developed for soft tissue reconstruction as a valid alternative to autologous fat grafting due to several problems. These include the generation of acidic degradation byproducts, which can trigger local inflammation and fibrotic encapsulation. Moreover, reproducing the viscoelastic “softness” of native adipose tissue (2–10 kPa) remains technically challenging. In fact, most synthetic materials are either too rigid to support adipogenesis or too fragile to maintain structural integrity over time after injection. Even when biofunctionalized, these scaffolds often elicit M1 macrophage-dominant immune responses, resulting in limited vascular integration and progressive volume loss. Finally, regulatory approval for long-term injectable polymers remains complex and costly, as it requires extensive evaluation of controlled degradation and long-term biocompatibility.

### 2.3. Adipose ECM-Derived Injectable Scaffolds

Adipose ECM-derived injectable scaffolds (i.e., decellularized adipose tissue (DAT), AAM, adipose-derived matrix, and decellularized adipose matrix (DAM)) are fabricated by removing cellular components from autologous, allogeneic or xenogeneic adipose tissue. The decellularization process typically combines physical disruption (e.g., freeze–thaw cycles or mechanical agitation) with chemical and enzymatic treatments, effectively preserving most of the biochemical composition and ultrastructure of the native ECM [58]. This multi-step process, lasting approximately 5–8 days, leads to adipose ECM-derived injectable scaffolds suitable for allograft transplantation that are rich in collagen, laminin, fibronectin, glycosaminoglycans and growth factors [59]. In principle, ECM-derived hydrogels can also be obtained from many other soft tissues [60]. However, depending on the tissue source, they exhibit specific differences. In this context, it should be noted that adipose ECM-derived injectable scaffolds contain significant levels of the α4 chain of laminin 411, which may be explained by the dense microvascular networks in fat tissue [23]. Moreover, they are characterized by high levels of FGF-1 and FGF-2, which are, for instance, much greater than those of tumor-derived Matrigel™ [61].

In 2011, Young et al. first described a reproducible protocol capable of generating an injectable DAT derived from human fat that effectively preserves adipose-specific ECM cues and mechanical integrity [22]. More recently, enzyme-free and supercritical carbon dioxide-based decellularization techniques have been developed to better retain native growth factors and vesicle-bound proteins compared with conventional detergent-based methods [62]. Despite significant advancements over the past decades, no standardized protocol to obtain adipose ECM currently exists. In fact, different laboratories employ various detergents, enzymes and delipidation steps, resulting in a high variability of the final scaffold bioactivity [63] (Table 3).

A growing body of evidence demonstrates that ECM-derived injectable scaffolds support adipogenesis and vascularization, both in vitro and in vivo. In vitro, these materials retain key structural proteins as well as growth factors, providing a substrate that directs ASC differentiation more efficiently than type I collagen scaffolds [58,69]. In vivo, Young et al. [66] observed that DAM hydrogels, crosslinked with transglutaminase and seeded with ASCs, markedly enhance neovascularization and adipose tissue formation 4 weeks after implantation into nude mice when compared to DAM alone or a HA-derived commercial filler. Similarly, Han et al. [70] reported a more pronounced formation of well-vascularized adipose tissue 8–12 weeks after implantation of DAT scaffolds pre-seeded with ASCs in rats when compared to unseeded scaffolds. Furthermore, Zhang et al. [71] showed that FGF-2 incorporated into DAM significantly improves adipogenesis in mice, achieving an adipocyte density after 12 weeks which is comparable to that of native fat. The effective replacement of DAM with well-vascularized adipose tissue likely reflects its capacity to induce differentiation in up to 90% of encapsulated ASCs [65]. Mechanically, adipose ECM hydrogels exhibit remarkable stability, especially when crosslinked. They maintain structural integrity even after multiple freeze–thaw cycles, thus supporting clinical translation [64].

More recently, Anderson et al. [68] tested an injectable human DAT from bench to bedside. In vitro, the scaffold was able to enhance ASCs migration, adhesion and adipogenic differentiation, while exhibiting a volume retention comparable to autologous fat grafts but without cysts, necrosis or calcifications. In immunocompromised mice, the DAT maintained ~60% of its initial volume over 12 weeks. Histological analyses revealed a progressive replacement of the scaffold with newly formed adipose tissue. In a porcine model, injection volumes up to 20 mL demonstrated dose-dependent volume stability and minimal immune response, with implants remaining visible and well-integrated at 4 weeks. In an additional phase 1 human trial, small subcutaneous DAT implants (1–4 mL) were excised after 1–18 weeks, showing excellent safety with no adverse immune reactions, and progressive cellular infiltration and vascularization over time [68].

The first FDA-approved human adipose ECM-derived injectable scaffold is Renuva^®^ (MTF biologics, Edison, NJ, USA). This injectable AAM allows cell ingrowth and gradually remodels into native fat, showing favorable integration and volume retention over several months in vivo [67,72,73,74]. The first human trial with Renuva^®^ was conducted in 2019 by Kokai et al. [67]. For this purpose, 2.5–5.5 mL of the AAM was injected into the dorsum of the wrist of 15 patients using a 19-gauge blunt-tip cannula after local anesthesia. The injectable scaffold maintained soft-tissue volume up to 4 months, especially when 4.5 mL or more volume was injected, becoming palpably similar to adipose tissue. Despite initial pain and injection site redness and swelling across most patients, no major adverse events (i.e., infections, allergic reactions and/or immune responses) were detected [67]. The same group evaluated the AAM in the pannus of 10 patients 3 or 6 months before scheduled abdominoplasty. For this purpose, they injected 120 mL of the AAM in 6 different sites (20 mL each). By this, they demonstrated that the scaffold was safe, biocompatible and adipogenic and integrated well into the surrounding host tissue without inducing inflammation. An increased density of adipocytes was observed already 3 months after injection, and remodeling of the scaffold was complete after 6 months, maintaining the original grafted volume [72].

**Figure 3:**
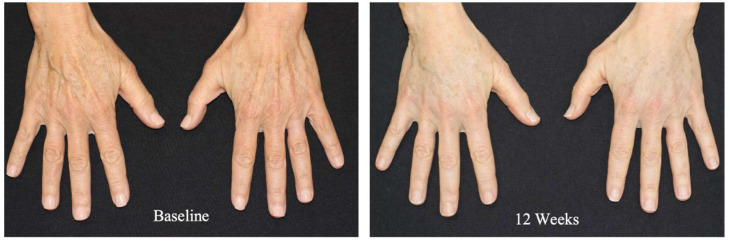
Allograft adipose matrix injection (2 × 1.5 mL) into the dorsal hands of a 67-year-old woman (Photograph courtesy of Joel Cohen MD). Reprinted with permission from [74] under the terms of the Creative Commons Attribution License.

Gold et al. [73] conducted a multicenter, prospective open-label study assessing Renuva^®^ for bilateral temporal hollowing. Ten subjects received ≤3 mL per side injected subcutaneously into the temporal fossa. At 24 weeks, the volume retention rate was ~75%. Histology demonstrated progressive neovascularization, host cell infiltration and adipose remodeling without either inflammatory or fibrotic reactions. Hence, this study confirmed Renuva^®^ as a safe, off-the-shelf alternative to autologous fat grafting with comparable short-term volumetric retention but without donor-site morbidity. Subsequently, Gold et al. [74] reported a multicenter real-world series expanding the use of this AAM to the midface, dorsal hands and post-lumpectomy breast asymmetry, with injection volumes ranging from 1 to 3 mL (Figure 3). At 12 weeks, significant improvements were observed in Mean Midface Volume Severity Assessment (MMVSA) and Global Aesthetic Improvement Scale (GAIS) scores, with high patient satisfaction and no serious adverse events. The authors highlighted practical advantages, including ease of injection and natural outcomes. Likewise, Leneva^®^, another processed human AAM-based filler from the same manufacturer, has demonstrated utility in soft tissue reconstruction beyond cosmetic applications. In fact, Shanin et al. [75] reported that intradermal injection of Leneva^®^ into recurrent diabetic foot ulcers reduces plantar pressure by ~70% and resolves pre-ulcerative callus within 4 weeks, acting as a biologically active “cushion”.

In summary, adipose ECM-derived injectable scaffolds show great integration into the host tissue, closely mimicking the native structure and bioactivity of adipose tissue without the need for further modification. They support both adipogenesis and angiogenesis while minimizing inflammatory and fibrotic responses. Clinically, these scaffolds have been applied as an off-the-shelf adipose tissue alternative for breast volume restoration, lipoatrophy and congenital defects, achieving good integration and volume retention up to 12 months [72,73,74,75,76].

## 3. Conclusions

Injectable scaffolds closely mimicking native adipose tissue may represent a viable alternative to achieve a “like with like” reconstruction of soft tissue defects. By overcoming the limitations associated with autologous fat grafting, such as unpredictable graft resorption rates and donor site morbidity, these systems offer a minimally invasive and biologically favorable alternative for volume restoration.

**Figure 4:**
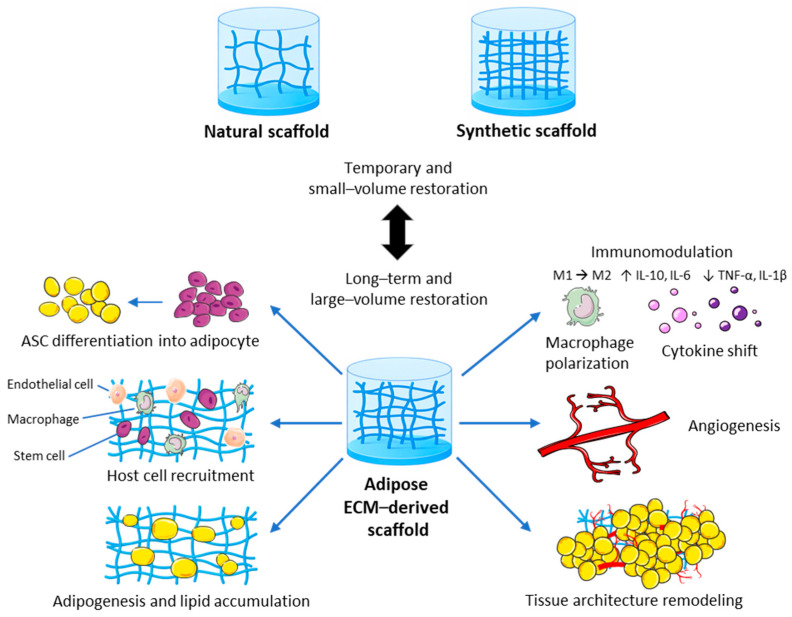
Advantages of adipose ECM-derived injectable scaffolds compared to natural and synthetic scaffolds. Adipose ECM-derived scaffolds may enable long-term, large-volume tissue restoration by retaining tissue-specific biochemical and mechanical properties. They can promote ASC differentiation into adipocytes, host cell recruitment, adipogenesis with lipid accumulation, immunomodulation via M2 macrophage polarization, angiogenesis, and remodeling of adipose tissue architecture. M = macrophage; ↑ = increased; ↓ = reduced.

Among the various scaffold types, adipose ECM-derived injectable scaffolds currently exhibit the greatest translational potential owing to their intrinsic biocompatibility, angiogenic capacity and ability to remodel into functional adipose tissue. These scaffolds not only restore volume but also actively participate in tissue regeneration through the recruitment of progenitor cells and the modulation of the local immune microenvironment (Figure 4). Nonetheless, the field of soft tissue engineering still faces some challenges and further progress is required to spread regulatory approval. For example, the establishment of standardized manufacturing protocols and donor pooling systems as well as robust long-term clinical data may ensure reproducibility. As patients increasingly opt for nonsurgical procedures that offer predictable results, the development of minimally invasive adipose tissue substitutes, such as injectable scaffolds, has become increasingly important. For this aim, future research should focus on optimizing large-volume, long-lasting and immunologically safe injectable adipose substitutes. If this succeeds, the next generation of injectable scaffolds may redefine current reconstructive surgery techniques by providing a readily available alternative to conventional autologous fat grafting.

## Figures and Tables

**Table 1:** Overview of the properties of natural, synthetic and adipose extracellular matrix (ECM)-derived injectable scaffolds for soft tissue reconstruction, as outlined in the present review article.

Injectable Scaffold	Bioactivity	Biocompatibility	Inflammatory and Fibrotic Reaction	Mechanical Strength
Natural	++	++	+	+
Synthetic	+	+	+++	+++
Adipose ECM-derived	+++	+++	+	+

+ = low; ++ = moderate; +++ = high.

**Table 2:** Overview of natural-derived and commercially available fillers for soft tissue augmentation.

Commercial Name (Company)	Composition	Approval
Algeness^®^ (Advanced Aesthetic Technologies, Brookline, MA, USA)	Agarose gel	CE-marked
Bellafill^®^ (Suneva Medical, San Diego, CA, USA)	Type I bovine collagen gel	FDA-approved
Belotero^®^ (Merz Aesthetics, Frankfurt am Main, Germany)	HA gel	CE-marked and FDA-approved
Evolence^®^ (ColBar LifeScience Ltd., Herzliya, Israel)	Type I porcine collagen	CE-marked and FDA-approved
GeneFill DX (BioScience GmbH, Radeberg, Germany)	HA gel	CE-marked
Lava^®^ (BioScience GmbH, Radeberg, Germany)	Type I porcine collagen	CE-marked
Linerase^®^ (Euroresearch, Milan, Italy)	Type I equine collagen	CE-marked
Juvéderm^®^ (Allergan, Irvine, CA, USA)	HA gel	CE-marked and FDA-approved
Restylane^®^ (Galderma, Zug, Switzerland)	HA gel	CE-marked and FDA-approved
Revanesse^®^ VersaTM (Prollenium, Toronto, ON, Canada)	HA gel	CE-marked and FDA-approved
Saypha^®^ (Croma Pharma, Leobendorf, Austria)	HA gel	CE-marked
Sisderm^®^ (Euroresearch, Milan, Italy)	Type I equine collagen	CE-marked
Stylage^®^ (Vivacy, Archamps, France)	HA gel	CE-marked
Teosyal^®^ (Teoxane, Geneva, Switzerland)	HA gel	CE-marked and FDA-approved
YVOIRE^®^ (LG Chem, Seoul, South Korea)	HA gel	CE-marked
Zyderm^®^ (Allergan, Irvine, CA, USA)	Type I bovine collagen	FDA-approved

CE = Conformité Européenne; FDA = Food and Drug Administration; HA = hyaluronic acid.

**Table 3:** Overview of adipose ECM-derived injectable scaffolds, including the reagents used for their production as well as their in vitro and in vivo properties.

Tissue Source	Decellularization Reagent	Delipidization Reagent	Solubilization Reagent	In Vitro Property	In Vivo Property	Reference
Human	1% SDS, or 2.5 mM sodium deoxycholate	2.5 mM sodium deoxycholate with 500 U lipase and 500 U colipase	1 mg/mL pepsin, 0.1 M HCl	ASC adhesion, viability and proliferation ↑	Shape ↔	[22]
Human	3% peracetic acid, 1% Triton X-100, 2 mM EDTA, 600 U/mL DNase, 10 mM MgCl_2_	Triton X-100, extensive washing	1 mg/mL pepsin, 0.1 M HCl	ASC adhesion, viability and adipogenic differentiation ↑	Adipogenesis ↑ Inflammation ↔ Vascularization ↑	[64]
Porcine	2 U/mL dispase II, 50 mM Tris-HCl, 2 mM NEM, 8 mM EDTA	Extensive washing, centrifugation, 0.2 mm filtration	1% pepsin, 0.5 M acetic acid	ASC infiltration, TNF-α, MCP-1 expression ↑	Adipogenesis ↑ Vascularization ↑	[65]
Human	1% SDS, 0.01% Triton X-100	2.5 mM sodium deoxycholate with 500 U lipase and 500 U colipase	1 mg/mL pepsin, 0.1 M HCl	ASC adhesion, viability and adipogenic differentiation ↑	Not tested	[66]
Human	sodium deoxycholate, peracetic acid	1-propanol	1 mg/mL pepsin, 0.1 M HCl	ASC adhesion, infiltration and adipogenic differentiation ↑	Adipogenesis ↑ Biocompatibility ↔ Vascularization ↑ Volume ↔	[67]
Human	3% peracetic acid, 1% Triton X-100, 2 mM EDTA	Infinity TG Reagent (Thermo Fisher Scientific)	4 M guanidine HCl	ASC migration, adhesion and adipogenic differentiation ↑	Adipogenesis ↑ Necrosis, cysts ↓ Pro-regenerative environment ↑ Volume ↔	[68]

ASC = adipose-derived stem cell; MCP = monocyte chemoattractant protein; SDS = sodium dodecyl sulfate; ↑ = increased; ↓ = reduced; ↔ = unchanged.

## Data Availability

No new data were created or analyzed in this study.
